# Invasive Paget's Disease of the Breast: A Staging Pitfall Mimicking pT4 Disease

**DOI:** 10.7759/cureus.110432

**Published:** 2026-06-08

**Authors:** Anila Sharma, Raja Langer, Prerna Chadha, Gurudutt Gupta, Shruti Singh

**Affiliations:** 1 Pathology and Laboratory Medicine, Rajiv Gandhi Cancer Institute and Research Centre, Delhi, IND; 2 Surgery, Government Medical College, Jammu, Jammu, IND; 3 Pathology, Rajiv Gandhi Cancer Institute and Research Centre, Delhi, IND

**Keywords:** breast, breast staging, invasive paget's disease, mammary paget's disease, nipple ulcer

## Abstract

Invasive Paget's disease of the breast is a rare entity with therapeutic challenges. Herein, we report three patients with Paget's disease of the breast with dermal invasion, of which two cases had coexistent invasive breast carcinoma, while the third had an extensive in situ component. Case 1 was a 49-year-old lady presenting with nipple ulceration and discharge along with concomitant multicentric right breast lesions detected on mammography. Breast conservation surgery was performed which revealed extensive high-grade ductal carcinoma in situ on histopathology, associated with invasive Paget's disease. The patient completed adjuvant radiotherapy and shows no evidence of disease at present, four months following the completion of therapy. Case 2 was a middle-aged woman of 45 years, who was detected with multiple hypoechoic space-occupying lesions in the right breast on ultrasonography. Modified radical mastectomy was performed which showed a tumor-infiltrating lymphocyte-rich breast carcinoma along with invasive Paget's disease. Following surgery, she is currently on adjuvant chemotherapy with no evidence of disease. Case 3 was a 47-year-old lady presenting with a lump in the right breast with nipple ulceration. Microscopic examination of the modified radical mastectomy specimen showed a high-grade invasive breast carcinoma along with invasive mammary Paget's disease. Follow-up imaging showed generalized lymphadenopathy. The patient was advised adjuvant chemotherapy; however, she was lost to follow-up after receiving the first cycle of treatment. We present three cases of invasive Paget's disease of the breast highlighting the importance of recognition of this rare entity with distinct therapeutic implications.

## Introduction

Paget's disease of the breast (PDB) is an intraepidermal proliferation of neoplastic glandular cells in the nipple-areolar complex (NAC) without any breach of the basement membrane. It is a rare, albeit well-described, entity, accounting for 1-3% of primary breast carcinomas [1]. The clinical presentation of PDB is nipple erosion or ulceration which may be accompanied by nipple discharge. The pathogenesis of PDB involves two major theories. The first theory involves the retrograde extension of an underlying in situ carcinoma into the epidermis via the lactiferous ducts of the nipple, while the second theory suggests an intraepidermal origin from Toker cells, which are native cells of the nipple.

PDB is considered equivalent to ductal carcinoma in situ (DCIS) and staged as Tis as per the American Joint Committee on Cancer (AJCC) staging and is commonly associated with an underlying invasive carcinoma or DCIS [2,3].

Invasion of Paget's cells into the underlying dermis across the basement membrane is labelled as invasive PDB, and these infiltrative cells or nests show a clear separation from any underlying invasive breast carcinoma, if present. Invasive PDB is an extremely rare condition with fewer than 50 cases reported in the indexed literature so far [4-7]. Axillary nodal metastasis in exclusive invasive PDB is extremely uncommon with six reported cases thus far [5].

Invasive PDB is a staging trap that mimics pT4 disease but behaves biologically like an early disease, highlighting the importance of distinguishing between the two. pT4 disease is characterized by an invasive carcinoma causing ulceration of the overlying skin including the epidermis, whereas invasive PDB generally shows a superficial invasion into the dermis.

Histopathology with immunohistochemistry plays a key role in diagnosis. Cells of PDB express low-molecular-weight keratins (like cytokeratin 7 (CK7)) and show HER2/neu positivity in up to 90% of the cases. Herein, we present three cases of invasive PDB, one of which showed HER2/neu negativity.

## Case presentation

Case 1

A 49-year-old lady presented with nipple erosion and bloody discharge of three years' duration.

Ultrasonography (USG) showed cutaneous thickening with increased vascularity involving the right nipple along with bilateral mild subareolar and periareolar ductal prominence. The right breast showed fine pleomorphic calcifications in the subareolar and periareolar breast parenchyma. Multifocal right breast lesions were detected involving the upper outer and upper inner quadrants, measuring 1.8 cm (spiculated mass) and 0.4 cm in maximum dimensions, respectively. Breast Imaging Reporting and Data System (BI-RADS) score 5 was assigned to the right breast lesions, while the left breast was assigned a BI-RADS score of 2.

The patient underwent right breast conservative surgery and sentinel lymph node biopsy. Gross examination of the breast lump (10.5×11×3.5 cm) did not show any well-defined tumor; however, an ill-defined grey-white firm lesion was noted, measuring approximately 4.5 cm in the largest dimension. The NAC appeared ulcerated.

**Figure 1:**
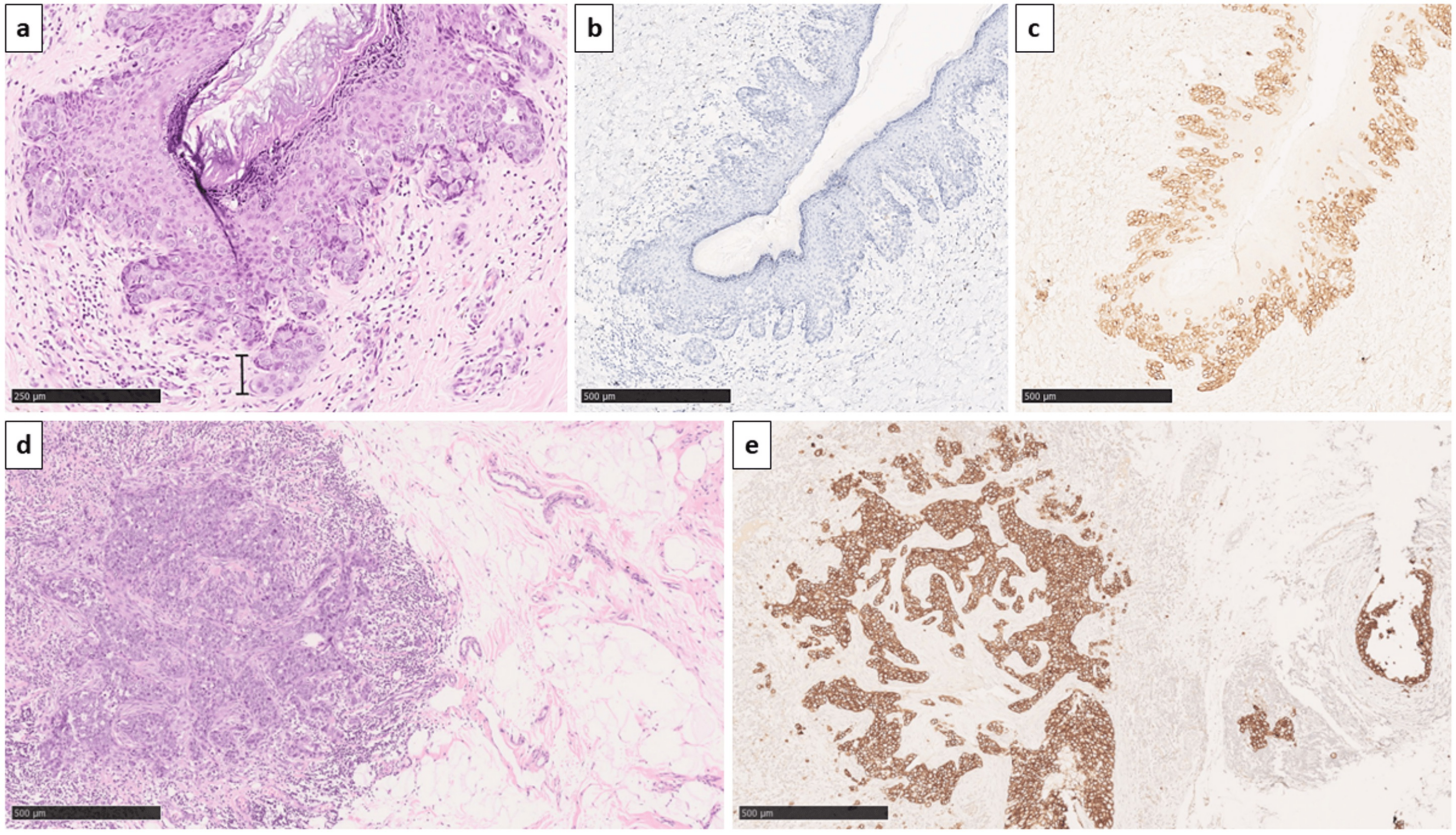
Case 1 (a) H&E section showing epidermal involvement by Paget's cells characterized by large pale-staining cells with prominent nucleoli within the squamous epithelium with superficial dermal invasion (invasive Paget's) annotated by a black bar. Extensive high-grade ductal carcinoma in situ with focal microinvasion. (b) Negative immunostaining for estrogen receptor. (c) HER2/neu immunohistochemistry showing strong membranous staining in intraepidermal Paget's cells. (d) H&E demonstrating the focus of microinvasive carcinoma. (e) HER2/neu immunoreactivity in the microinvasive carcinoma component and associated ductal carcinoma in situ.

On microscopy, extensive high-grade DCIS was seen in varied architectural patterns (comedo, papillary, and solid) with a single focus of microinvasion (<1 mm). The NAC showed invasive PDB with a horizontal extent of 12 mm and a depth of stromal invasion of <1 mm. All margins were free of DCIS. The six largest sentinel lymph nodes isolated were free of tumor. Pathological staging of pT1miN0 was rendered. On immunohistochemistry, the tumor was estrogen receptor (ER) and progesterone receptor (PR) negative, while HER2/neu was positive (score: 3+). Invasive PDB showed a similar immunoprofile. Figure 1 shows the histopathology and immunohistochemistry images for this case.

She underwent adjuvant short-course image-guided radiation therapy of 26 Gy in five fractions followed by 10 Gy in five fractions. The patient remains disease-free four months from the completion of radiotherapy.

Case 2

A 45-year-old woman presented with a serous nipple discharge and right breast lump for four months which was painful and progressive in size. USG showed multiple hypoechoic space-occupying lesions in the right breast varying in size from 8 mm to 17 mm in maximum dimension, involving the upper outer and upper inner quadrants. The NAC appeared unremarkable on imaging. Magnetic resonance imaging (MRI) of the right breast revealed altered signal intensity, irregular mass lesions involving the 2 o'clock and 3 o'clock positions along with linear ductal non-mass enhancement with nipple involvement (MR BI-RADS 5). Also, another well-defined lobulated enhancing retroareolar lesion (BI-RADS 4) was detected. The left breast appeared unremarkable (BI-RADS 1). Positron emission tomography (PET) scan showed only locoregional disease with breast masses and right axillary lymphadenopathy.

**Figure 2:**
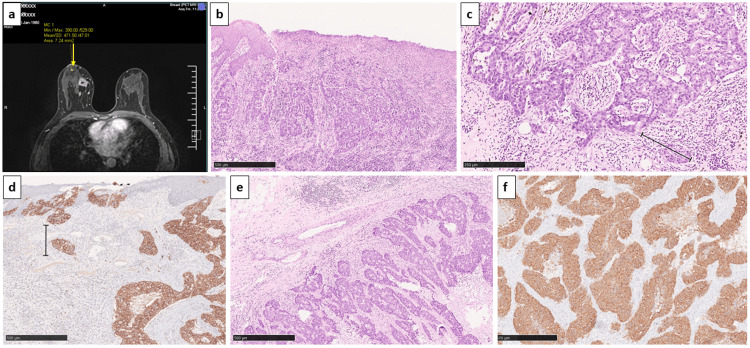
Case 2 (a) Magnetic resonance imaging demonstrating a lesion in the nipple with an underlying parenchymal lesion in the right breast (nipple lesion arrow marked). (b) H&E demonstrating the epidermal spread of Paget's cells within the nipple-areolar complex with associated ulceration. (c) Focus of invasive Paget's annotated by a black bar. (d) HER2/neu immunohistochemistry showing strong membranous staining in Paget's cells; black bar annotation shows the depth of dermal invasion. (e) H&E section showing invasive breast carcinoma, with tumor-infiltrating lymphocyte-rich stroma. (f) HER2/neu positivity in the invasive carcinoma.

The patient underwent a modified radical mastectomy. Macroscopic examination revealed two tumor foci, one in the upper outer quadrant (2.2×1.5×1.3 cm) and another in the retroareolar region (1.8×1.2×0.9 cm). The nipple appeared eczematous. Microscopically, both tumors had similar histomorphology of a grade 3 tumor-infiltrating lymphocyte-rich invasive breast carcinoma with the presence of invasive carcinoma in the intervening breast parenchyma as well, implying a single tumor focus of 4.8 cm in greatest dimension. The nipple showed invasive PDB with a horizontal extent of 16 mm and a depth of invasion of <1 mm. The large lactiferous ducts did not show the presence of any DCIS. Pathological stage of pT2N0 was rendered. Hormone receptor tests showed an overlapping profile in both the invasive tumor focus and the invasive PDB. ER was low positive (2% of tumor cells), and PR was negative, while HER2/neu showed strong positive expression (score: 3+). Figure 2 shows the radiology and microscopic images for this case.

The patient is currently on adjuvant chemotherapy (Adriamycin-cyclophosphamide-paclitaxel) seven months post-surgery.

Case 3

**Figure 3:**
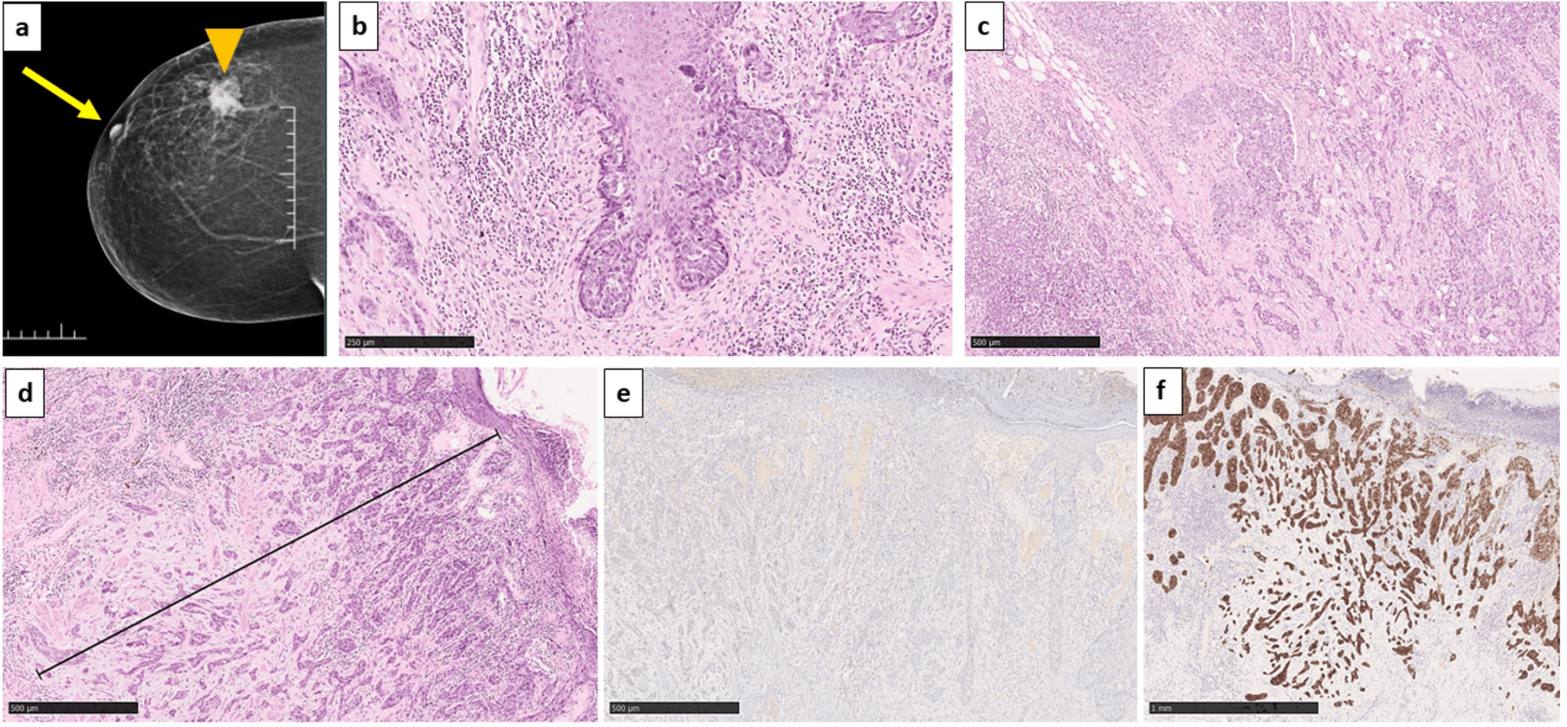
Case 3 (a) Mammogram demonstrating a lesion in the nipple (arrow) with a right breast mass (arrowhead). (b) H&E illustrating epidermal involvement by Paget's cells with invasive Paget's. (c) H&E showing invasive breast carcinoma. (d) H&E showing invasive Paget's with maximum depth of invasion into the dermis (annotated by a black bar). (e) Negative HER2/neu immunostaining in invasive Paget's. (f) Cytokeratin (AE1/AE3) immunohistochemistry highlighting intraepidermal and invasive Paget's.

A 47-year-old premenopausal lady presented with a lump in the right breast of three months' duration along with nipple ulceration. Mammography showed a 4 cm irregular, spiculated, high-density, right breast mass in the upper outer quadrant along with a few foci of cutaneous punctate calcifications. Histopathology revealed invasive breast carcinoma, no special type (NST), morphological grade 3, with the presence of invasive PDB involving the NAC. Invasive PDB measured 9 mm in horizontal extent and 2 mm in depth. One of the 13 axillary lymph nodes dissected was involved by tumor. A pathological stage of pT2N1a was assigned. The tumor was found to be triple negative on immunohistochemistry, and the PDB also showed a similar immunoprofile. Figure 3 shows the radiological and microscopic images for this case.

The patient was lost to follow-up after receiving one cycle of dose-dense adjuvant chemotherapy with Adriamycin, cyclophosphamide, and paclitaxel. She re-presented after 14 months from the completion of one cycle of chemotherapy, and follow-up PET-computed tomography (CT) was performed. It showed progressive disease in the form of supra- and infradiaphragmatic lymphadenopathy with hepatosplenomegaly and diffuse skeletal and marrow involvement. Posterior cervical lymph node biopsy confirmed nodal metastasis. Thereafter, the patient was again lost to follow-up.

Table 1 summarizes the key findings in all three cases.

**Table 1:** Clinicopathological profile of the three cases of invasive Paget's disease USG: ultrasonography; MRI: magnetic resonance imaging; DCIS: ductal carcinoma in situ; IBC NST: invasive breast carcinoma, no special type; BCS: breast conservation surgery; MRM: modified radical mastectomy; ER: estrogen receptor; PR: progesterone receptor; RT: radiotherapy

Cases	Age (y)	Clinical presentation	Radiology	Histopathology and pathological stage (pTNM)	Depth of invasion	Horizontal extent	Hormonal status	Treatment	Follow-up
1	49	Nipple erosion and bloody discharge × 3 years	On USG, cutaneous thickening with increased vascularity in the nipple. Multifocal spiculated mass	High-grade DCIS with microinvasive carcinoma and invasive Paget's pT1miN(sn)0	1 mm	12 mm	ER- PR- HER2/neu+ (3+)	BCS followed by adjuvant radiation therapy	Disease-free 4 months after the completion of RT
2	45	Serous nipple discharge with right breast lump × 4 months	On MRI, altered signal intensity, irregular mass along with non-mass enhancement with nipple involvement	IBC NST grade 3 with invasive Paget's pT2N0	1 mm	16 mm	ER LOW+ PR- HER2/neu+ (3+)	MRM followed by adjuvant chemotherapy	Currently undergoing chemotherapy 7 months post-surgery
3	47	Lump in the right breast with nipple ulceration × 3 months	On mammography, irregular spiculated mass with a few foci of cutaneous punctate calcification	IBC NST grade 3 with invasive Paget's pT2N1a	2 mm	9 mm	Triple negative	Adjuvant chemotherapy	Lost to follow-up --> progressive disease with distant metastasis --> lost to follow-up

## Discussion

PDB accounts for 1-3% of primary breast carcinomas [1]. Invasive Paget's disease with dermal invasion is an extremely rare occurrence, accounting for only 4-8% of all cases of PDB [4,8]. PDB may be overlooked or misdiagnosed as involvement of the overlying skin by invasive carcinoma, erroneously upstaging the tumor to pT4. Invasive PDB further accentuates the problem. The largest study of invasive PDB showed a mean age at diagnosis of 47.6 years (age range: 35-76 years) in all the reported patients. In their study, eczematous skin was the most common presenting complaint, coexistent with a palpable breast mass, which was due to underlying invasive breast carcinoma. DCIS was found in a few patients, while none of the patients were asymptomatic [4].

The three reported patients in this study were between 45 and 50 years of age, two of whom had an underlying invasive breast carcinoma (Cases 2 and 3), while one had extensive DCIS with microinvasion (Case 1). Case 3 showed invasive PDB which was negative for HER2/neu. This is contrary to previously reported cases in the literature [9,10].

Nipple involvement poses a diagnostic challenge for the radiologist, owing to difficulty in imaging, attributed to the location of the NAC, which is considered a "blind spot" for the radiologist [11]. Nevertheless, mammography is still the primary diagnostic modality, although it may be unable to detect any nipple abnormality. MR mammography can be of help in detecting underlying cancer but may not help in differentiating PDB from direct cutaneous extension, which mandates biopsy confirmation [12,13].

In Case 1, both mammography and USG picked up thickened NAC skin, while in Case 2, USG was unable to detect any lesion in the NAC. MRI of the breast proved to be superior in revealing nipple abnormality. In Case 3, mammographic examination detected cutaneous calcifications.

Clinically, all three patients had either excoriation, ulceration, or swelling of the NAC with or without an associated nipple discharge or breast lump. Surgery forms the mainstay of treatment, especially since PDB is commonly associated with an underlying invasive carcinoma, as seen in two of our cases as well.

One of the cases (Case 3) showed axillary nodal metastasis. This patient also showed disease progression 14 months after receiving one cycle of adjuvant chemotherapy.

The morphologic differential diagnoses for PDB include Bowen's disease, melanoma, and Toker cell hyperplasia. Immunohistochemistry can aid in resolving these differentials. Bowen's disease shows high-molecular-weight cytokeratin expression apart from the markers of squamous differentiation like p40, whereas PDB shows low-molecular-weight cytokeratin (CK7) positivity instead. Melanoma is cytokeratin negative but positive for melanocytic markers such as S100, SOX10, Melan A, and HMB45, which are negative in PDB, similar to our cases. Toker cell hyperplasia forms a pertinent differential especially in cases with no underlying invasive breast carcinoma. Toker cells are devoid of any significant nuclear atypia unlike PDB but show an overlapping immunoprofile in the form of CK7 and ER positivity. However, HER2/neu expression, which is positive in up to 90% of PDB, is not seen in Toker cells [14]. This is in discordance with one of our cases (Case 3) wherein Paget's cells were HER2/neu negative. This can probably be explained by the immunoprofile of the associated invasive breast carcinoma, which was also triple negative, lending credence to the theory of retrograde spread of neoplastic cells via lactiferous ducts.

The challenge in cases of PDB remains staging, while the prognosis is governed by the stage of the underlying invasive carcinoma, commonly present. Invasive PDB is not associated with a poor prognosis, unlike locally advanced breast cancer with overlying skin involvement (pT4 disease). This necessitates the need for the distinction. Invasive PDB should not be regarded as pT4, as regional or distant metastasis is not seen in cases of dermal invasion by PDB [15]. Hence, most cases described in the literature were either T1mi or T1a [16].

Two cases in our study (Cases 2 and 3) were T2, while one case was T1mi apart from all having invasive PDB. Both the two cases with invasive breast carcinoma were staged as per the size of the primary invasive tumor in the breast parenchyma. The dilemma remains whether to label such tumors as multiple (m) tumors and to stage them as per the largest invasive tumor.

Skin invasion in breast carcinoma is a poor prognostic factor, whereas invasive PDB in itself is considered to have a favorable outcome [15]. Limited literature available on invasive PDB warrants further research to shed light on its clinical significance and management strategy [8,17].

There is limited literature regarding invasive PDB, and this case series attempts to explore the challenges encountered in this rare entity. We herein include one case of invasive PDB which showed HER2/neu negativity as opposed to the widely accepted HER2/neu-positive status reported in the literature.

This study is limited by a small sample size and a short follow-up period available in only two of the three cases reported.

## Conclusions

Invasive PDB is consistently associated with nipple ulceration, minimal dermal invasion, frequent HER2/neu positivity, imaging difficulty, and a high risk of erroneous pT4 upstaging. Recognition of this rare entity requires a diligent histopathological examination and immunohistochemistry/ancillary testing. Failure to recognize leads to management error.
